# Supplementary material for: Genome Analysis of Phytophthora nicotianae JM01 Provides Insights into Its Pathogenicity Mechanisms
Source: Plants (Basel). 2021 Aug 6;10(8):1620. doi: 10.3390/plants10081620 (PMC8400872; doi:10.3390/plants10081620)
Supplement: Supplementary file 1 [file plants-10-01620-s001.zip › Table S3 Elicitors in Ph.nicotianae and other species.pdf]

Table S3 Elicitors in *Ph. nicotianae* and other species

	CBEL	INF	OPEL	PEP
Achlya hypogyna	40	5	15	0
Albugo candida	3	6	6	4
Ectocarpus siliculosus	2	0	2	0
Fragilariopsis cylindrus	2	0	0	0
Hyaloperonospora arabidopsidis	2	9	3	3
Phytophthora capsici	16	29	14	18
Phytophthora cinnamomi	25	35	15	21
Phytophthora nicotianae	16	36	16	21
Phytophthora nicotianae race 0	9	20	8	5
Phytophthora parasitica	17	33	22	23
Phytophthora sojae	26	46	14	21
Phytophthora ramorum	21	35	11	19
Phytophthora infestans	19	32	32	12
Phaeodactylum tricornutum	0	0	0	0
Pythium aphanidermatum	40	25	12	8
Pythium arrhenomanes	53	26	13	8
Pythium insidiosum	17	41	11	29
Pythium irregulare	26	29	14	11
Pythium vexans	15	21	16	5
Pythium iwayamai	35	22	11	12
Pythium ultimum	34	31	15	9
Saprolegnia parasitica	50	5	20	0
Tetrahymena thermophila	0	0	0	0
Thraustotheca clavata	27	2	14	0
Thalassiosira pseudonana	0	0	0	0
