# Supplementary figures and images for: Genome Analysis of Phytophthora nicotianae JM01 Provides Insights into Its Pathogenicity Mechanisms
Source: Plants (Basel). 2021 Aug 6;10(8):1620. doi: 10.3390/plants10081620 (PMC8400872; doi:10.3390/plants10081620)

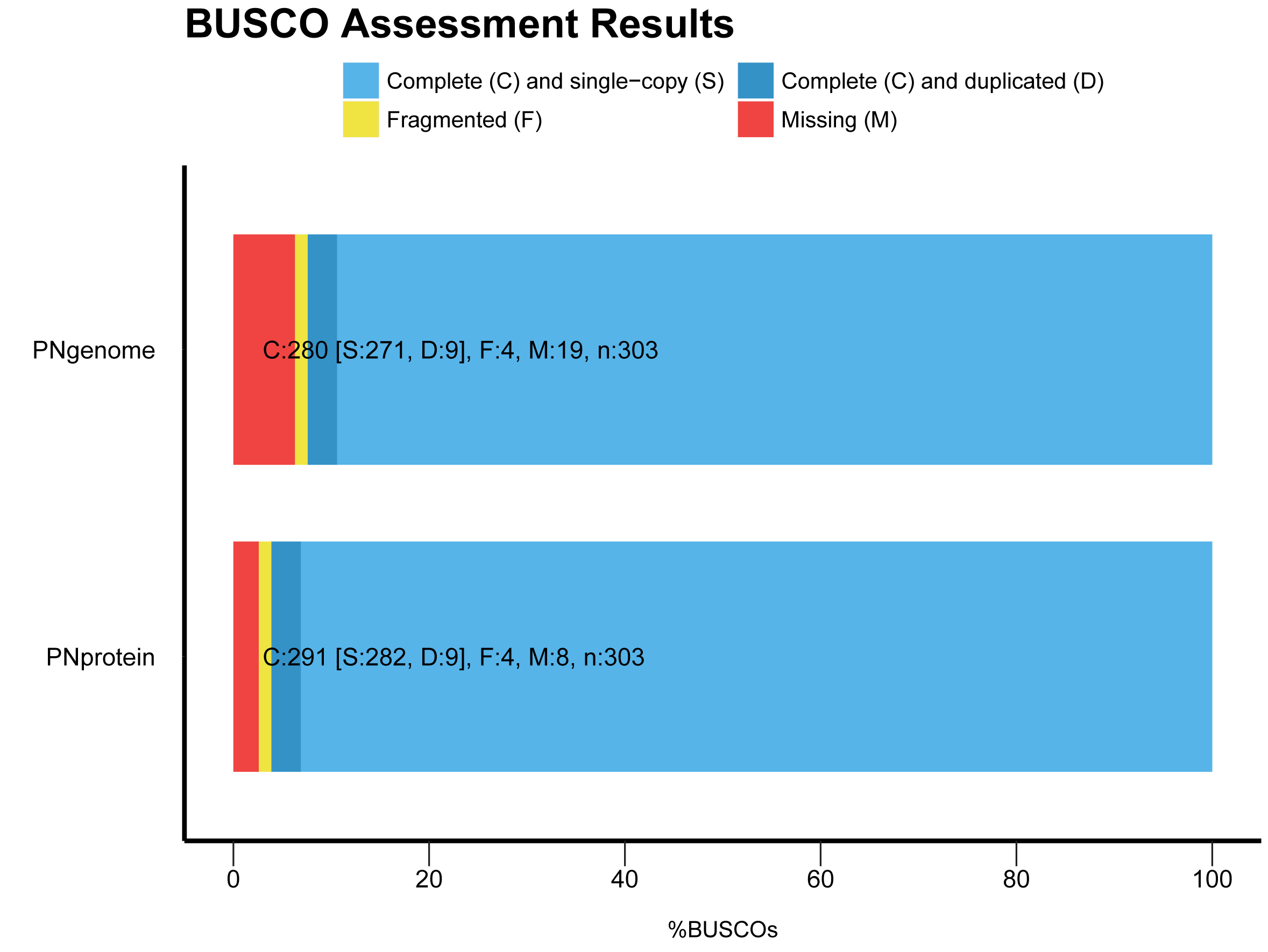

Supplement: Supplementary file 1 [file plants-10-01620-s001.zip › Figure S1 BUSCO assessment rsults of Ph. nicotianae.tif]

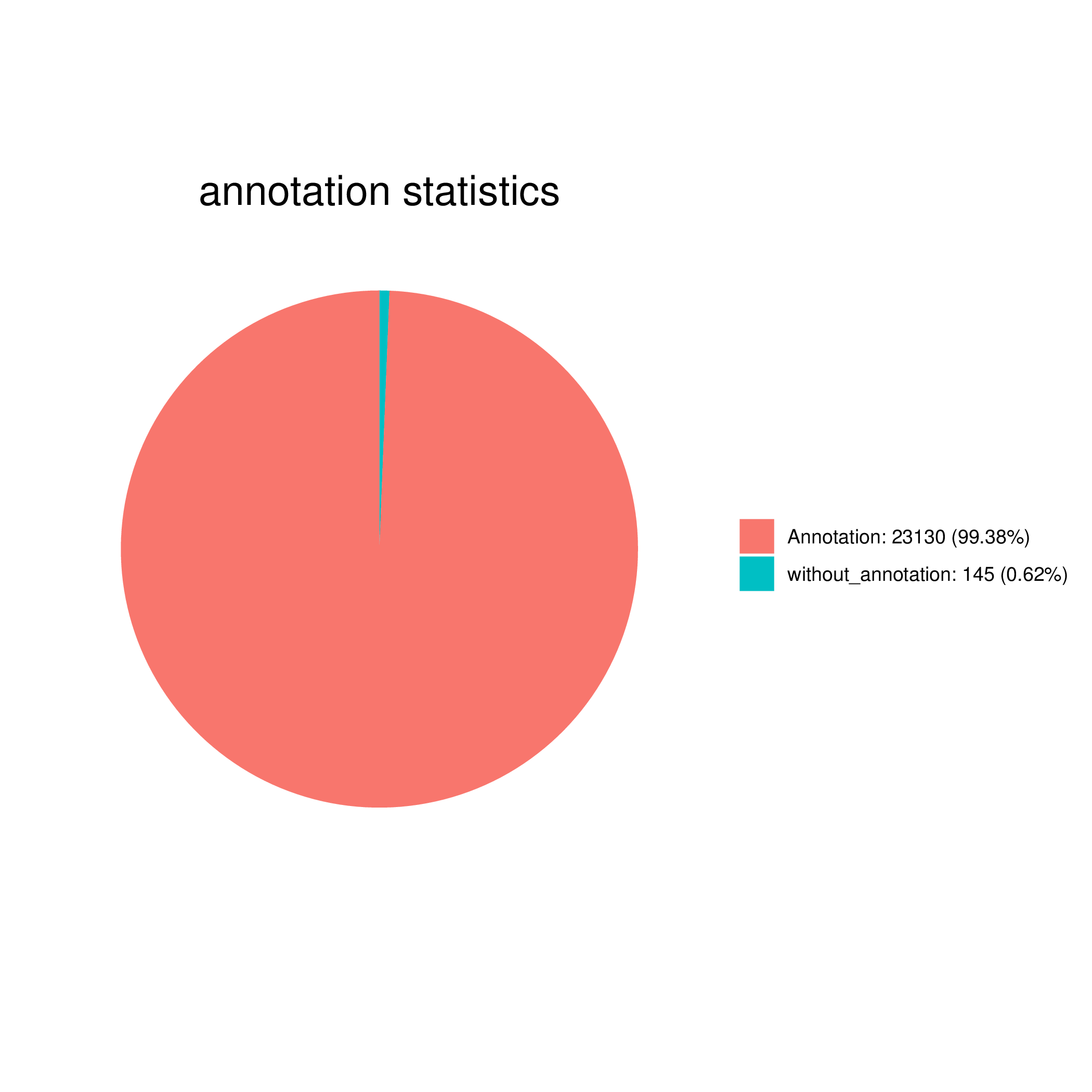

Supplement: Supplementary file 1 [file plants-10-01620-s001.zip › Figure S2 Nr blast results of Ph. nicotianae genome.png]

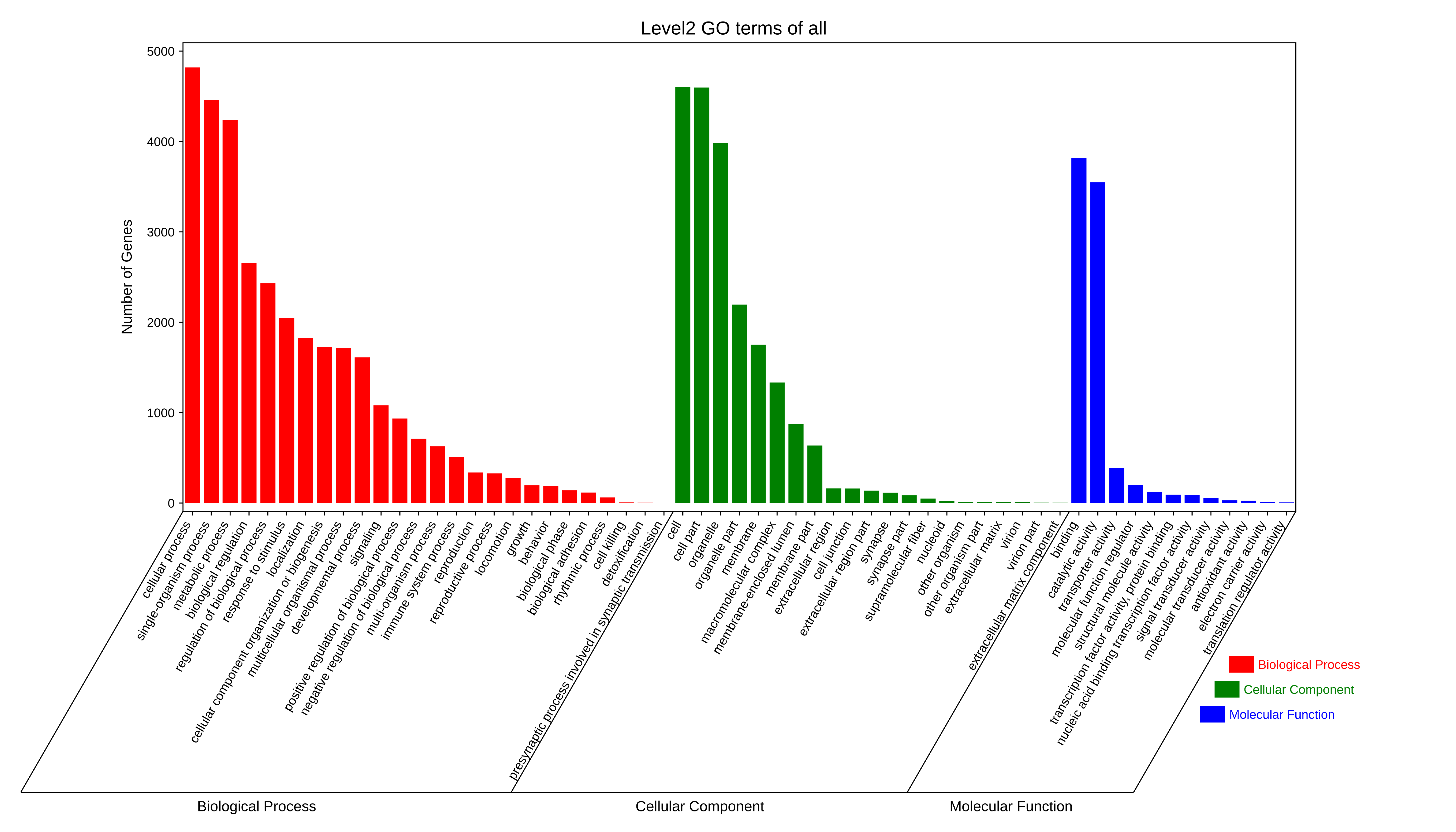

Supplement: Supplementary file 1 [file plants-10-01620-s001.zip › Figure S3 Overview of Go annotation with Ph. nicotianae genome.png]

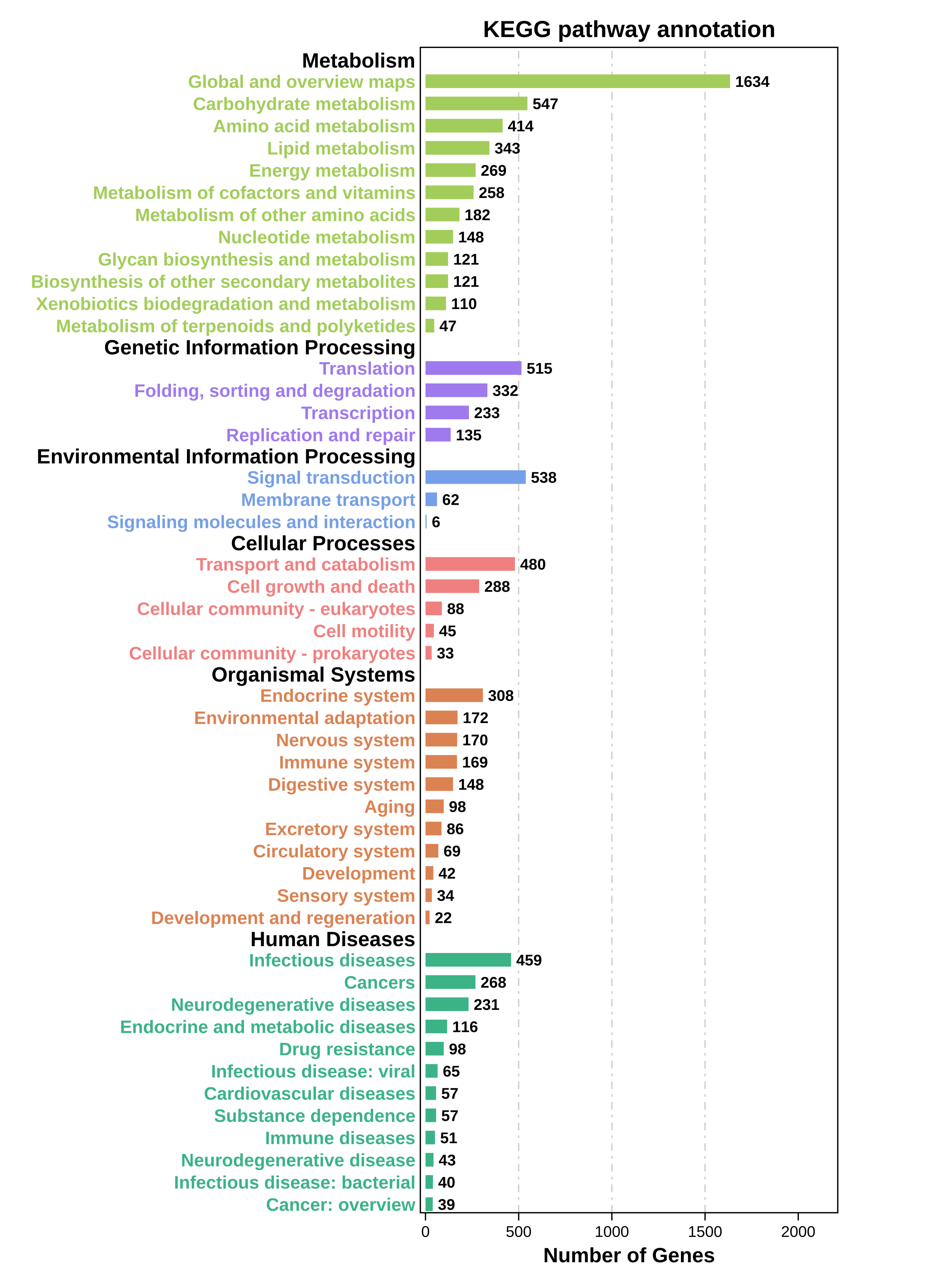

Supplement: Supplementary file 1 [file plants-10-01620-s001.zip › Figure S4 Barplot of KEGG annotation for Ph. nicotianae genome.png]

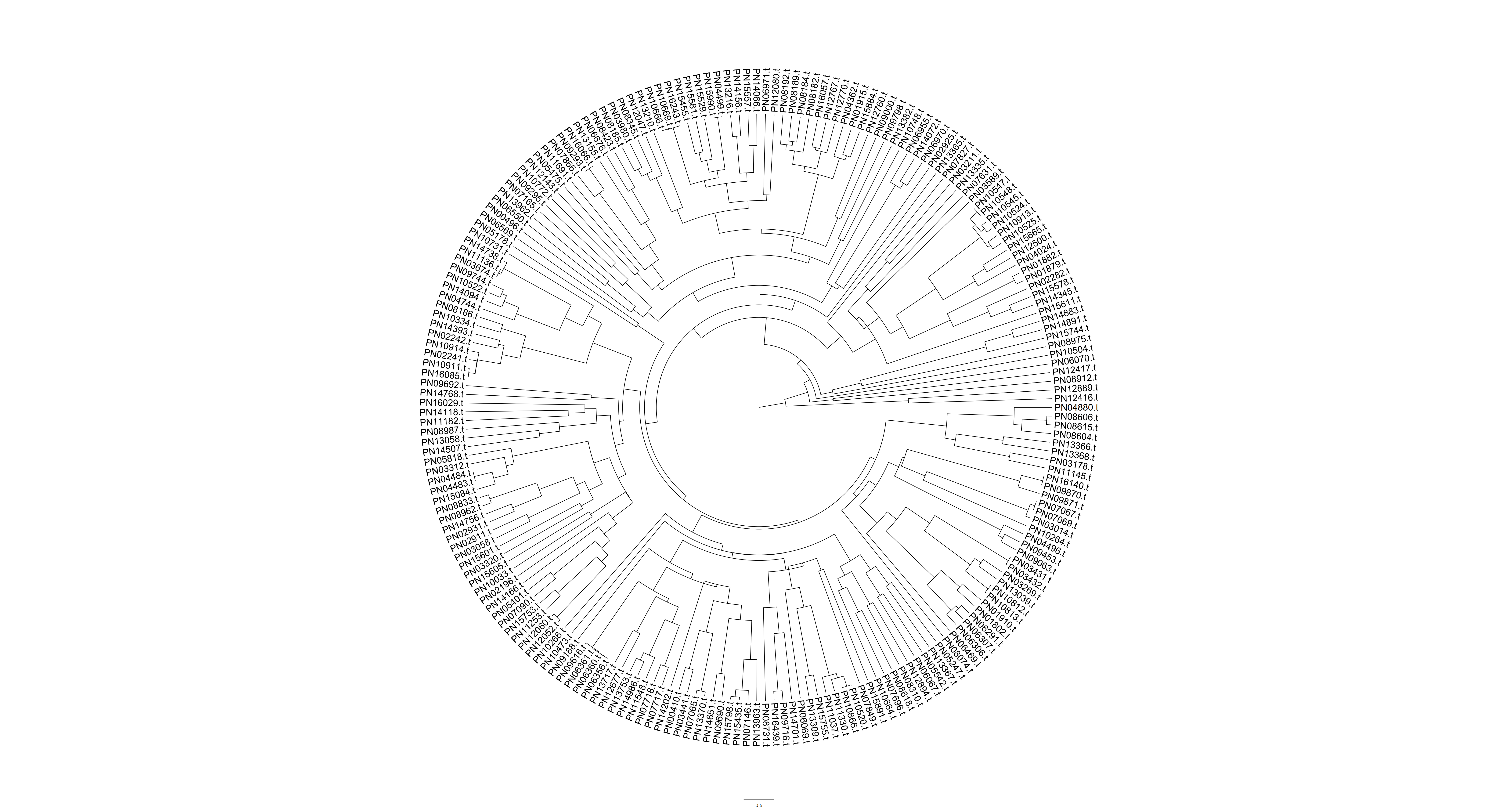

Supplement: Supplementary file 1 [file plants-10-01620-s001.zip › Figure S5 Phylogenetic tree for RXLR effectors in Ph. nicotianae.png]

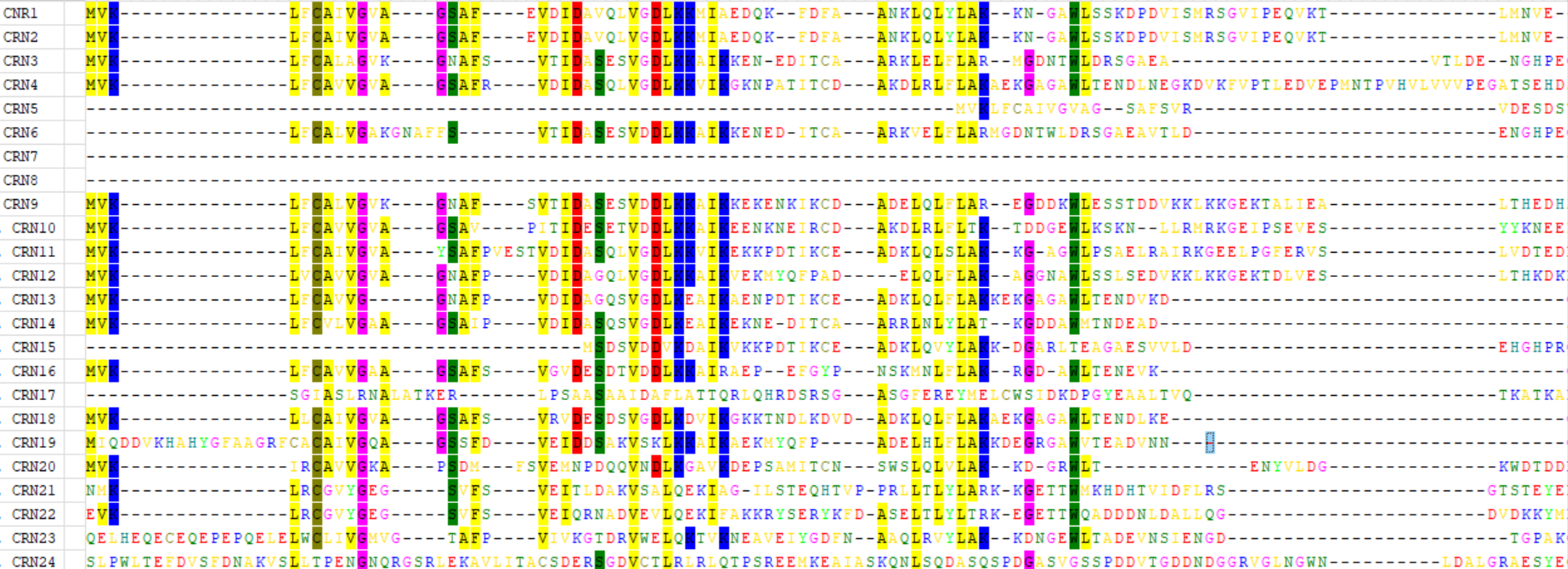

Supplement: Supplementary file 1 [file plants-10-01620-s001.zip › Figure S6 Conserved residues in the candidate CRN effector proteins in Ph. nicotianae .png]

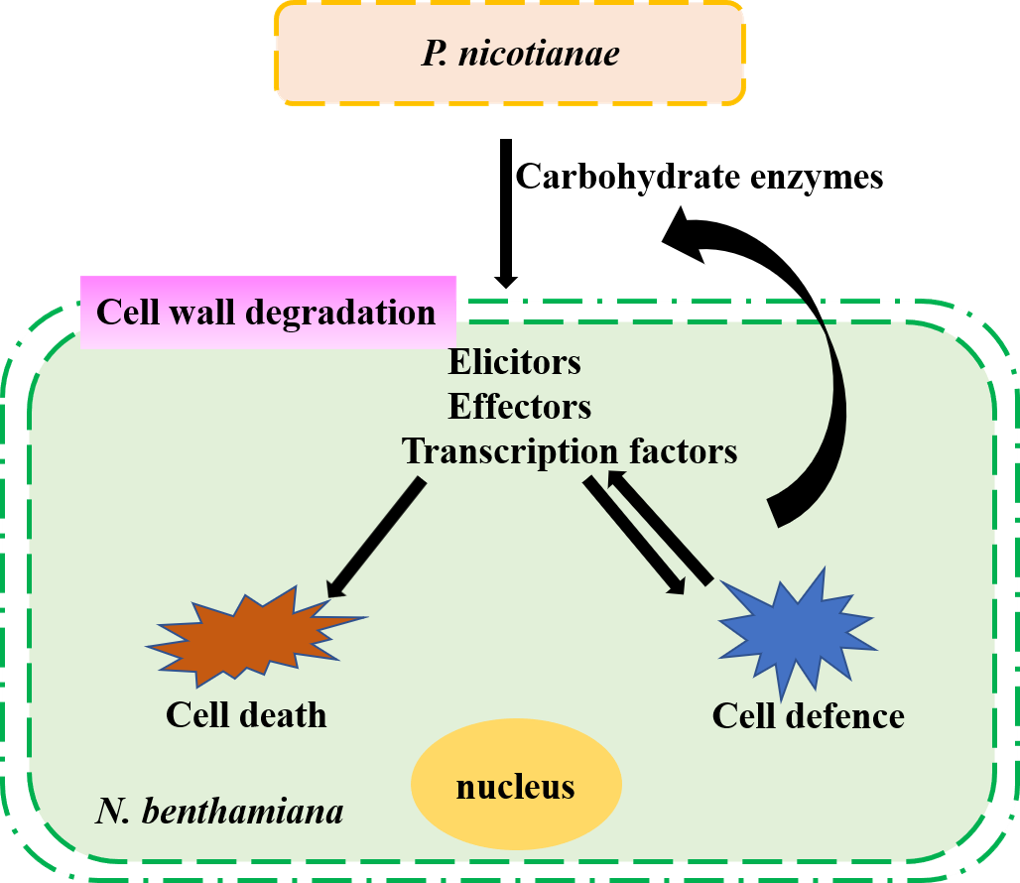

Supplement: Supplementary file 1 [file plants-10-01620-s001.zip › Figure S7 The predicted mechanism of Ph.nicotianae.tif]
